# Surgery, Obstetrics and Gynecology

**Published:** 1904-08

**Authors:** William Warren Potter

**Affiliations:** Buffalo


### Surgery, Obstetrics and Gynecology.

Conducted by WILLIAM WARREN POTTER, M. D., Buffalo.

### VERATRUM VIRIDE IN SURGERY.

C. L. Bonifield (*American Jour. of Obstetrics*) speaks very highly of veratrum in surgery. It relieves inflammatory pain and lowers the temperature. In appendicitis, after the principles taught by Ochsner have been applied—that of keeping the stomach and bowels emptied to arrest the peristaltic movements—veratrum viride meets every other indication for treatment. It sustains the heart by making it work easier; it stimulates elimination of the poison of the liver, kidneys and skin; it lowers the temperature and relieves the pain. It is possible that by its action upon the circulatory system it brings an army of leukocytes to the field of battle and in this way helps to destroy the invading army of germs. The circulation is much more profoundly influenced by veratrum than by hot and cold applications. In rapid acting hearts in post-operative peritonitis, after the bowels have been emptied, veratrum is a most valuable agent in slowing the heart's action. It is better than digitalis and strychnin, as it lessens the cardiac labor and gives time for rest between beats. Here, too, its eliminative action is most helpful. The preparation of the drug preferred by this author is Norwood's tincture. Where prompt action is wanted, as in eclampsia, it is best to give deep subcutaneous injections. Or, it may be given in this way in appendicitis, if it is desirable to keep the stomach empty. It produces some irritation at the point of injection, though the writer has never seen an abscess. The dose varies from five to thirty minims, according to the size and age of the patient. The toxicity of the drug has been overrated by the rank and file of the profession. H. C. Wood says that it is one of the safest of all the cardiac depressants. *Medical Standard.*

---

### PREGNANCY AND FORCEPS DELIVERY WITH UTERINE CANCER.

Dr. I. A. Shirley, Winchester, Ky., reported to the Kentucky State Medical Association (*Virginia Med. Semi-Monthly*) at its last meeting the case of Mrs. F., age 38 years, who had lost four small children by drowning. The shock, of course, was severe. Shortly afterward she found herself flooding freely and often from the vagina. On examination, unmistakable evidences of cancer of the cervix were found. He sent her to Dr. Chas. A. L. Reed, of Cincinnati, who concurred in the diagnosis, and advised immediate vaginal hysterectomy—stating that he considered it an ideal case for operation. On attempting the procedure a few days later, however, he found such involvement of the surrounding structures that he contented himself with removing a portion of the anterior lip of the cervix, and sent her home. After her return she became pregnant.

At term Dr. Shirley succeeded in dilating the os through the cancerous mass sufficiently to introduce high forceps, and delivered a good size girl baby that is now some eight or nine years old. Profuse hemorrhages came from many tears in the malignant growth, but these were finally checked by gauze tamponade of both the uterus and vagina. The mother recovered without special departure from the usual getting up, but died eighteen months later from a continuation of the disease.

The question is, did the severe shock of grief due to the drowning of her four children—as is believed by some—have anything to do with the starting of the malignant growth?

The case is worthy of record because of the occurrence of pregnancy and parturition at term in a woman whose cervix uteri had been removed.

---

### PROPHYLAXIS OF PUERPERAL SEPSIS.

George L. Broadhead (*Medical Record,* April 23, 1904,) says that puerperal sepsis is still a very common disease, except in hospital practice, where it has markedly decreased within the past few years. Statistics of mortality in private practice are extremely unreliable, because of the fact that death from sepsis is frequently ascribed to causes other than septic infection. It is said that in England—outside the lying-in hospitals—sepsis claims as many, or perhaps more, victims than it did twenty or forty years ago. If the vagina is free, as has been shown, from pathological bacteria, the cause of sepsis must necessarily be the introduction of septic material from without. This being the case, the question naturally arises: "is it necessary or advisable to make internal examinations during labor?" The object to be obtained in making such an examination is four-fold: We desire to inform ourselves as to (1) the presentation and engagement; (2) the position; (3) the degree of dilatation; and (4) the possible presence of a prolapsed cord. As to presentation, engagement and position, the diagnosis can almost invariably be made out by careful external examination. Many lives are lost every year from internal examinations which are absolutely unnecessary. As to dilatation, we should be guided largely by the character of the pains, their frequency, the duration of labor, and the parity of the patient. If she is to be left for any length of time dilatation must be taken into account; but if the physician is to remain with the patient, or is ready at any time to respond to the call, internal examination to determine the amount of dilatation is unnecessary. In the writer's own practice he has demonstrated over and over again that labor can be conducted successfully with no vaginal examination whatever.

If internal examination is necessary, the hands and vulva should be thoroughly sterilised. Chloride of lime, one teaspoonful and acetic acid, two teaspoonfuls, to the quart, is the strength used in sterilisation of the vulva; while for the hands it is used twice this strength—after using soap and water, of course. Gloves are recommended.—*St. Paul Med. Jour.*

---

### STERILITY DUE TO THE MAN.

E. H. GRANDIN (*Medical Record,* March 26, 1904, quoted also in the *Medical Age,*) emphasises the importance of gonorrhea and syphilis as etiological factors in the production of sterility, and enters a vigorous protest against present educational methods, by which young men are kept in ignorance of the wide-spread dangers attending the contraction of these diseases. Gonorrhea is responsible not alone for sterility, but also for many cases of pelvic disease in women and blindness in children. Man is responsible for race suicide in its true sense; in fully 45 per cent. of the cases of sterility in women the man is the responsible party. With syphilis, while a woman might conceive she as a rule miscarries, which practically amounts to sterility. The laity fails to realise and physicians do not lay sufficient stress upon the preponderant influence of the rôle played by men in the etiology of sterility in women. In every case of sterility the semen of the male should be examined, because although a man may be able to copulate he may be incapable of impregnation. In from 6 to 8 per cent. sterility is due to the fact that the man is sterile in that his semen does not contain spermatozoa, although he possesses the power to perform the sexual act. Every young man should be instructed as to the risk he runs when he contracts gonorrhea or syphilis, and the possible risk he carries to the woman whom he marries. By proper educational methods the operating gynecologist will be deprived of nearly 60 per cent. of his work, while on the other hand the human race will be immeasurably benefited.

---

### THE URINE IN PREGNANCY.

W. WILSON (*British Med. Jour.,* March 19, 1904,) discusses from experience the significance of the analysis of the urine of pregnancy with especial reference as a warning of impending eclampsia. As a general rule normal urinary findings are indicative of normal conditions in pregnancy, but abnormalities may exist in the specific gravity, urea excretion, and in the presence of albumin and glucose which are followed by normal labor. Of a considerable number of urine analyses during pregnancy only a small percentage showed entire freedom from albumin and glucose, but in no instance, provided there was no serious renal change, did the urine fail to return to normal after labor, except in a few cases where fatal eclampsia occurred. Small traces of albumin may be due to pressure by the gravid uterus causing renal congestion, but careful microscopic examination for granular and epithelial casts affords the only reliable guide as to the state of the kidneys themselves. The presence of glucose in small quantities is in itself of little moment as regards the outcome of the pregnancy, but when it represents a diabetic condition it is of grave import.

After advocating the periodical examination of the urine in all cases of pregnancy, and with greater frequency as term is approached, the author points out that the most dependable indications of impaired renal function and possible eclampsia are the presence of decided quantities of serum-albumin, the diminution in urea excretion, and the presence of casts, and the like. In young women even with normal urine the possibility of eclampsia must be borne in mind, and should it arise it may be equal in severity to those cases in which morbid conditions of the urine have given warning of its occurrence. Though not an unfailing guide, urine analysis affords the most trustworthy warning of danger that we possess, and in the event of eclampsia arising venesection and normal saline transfusion are the most reliable remedies.—*Medical Age.*

---

### SIGNIFICANCE OF URINALYSIS IN PREGNANCY.

WILSON (*American Jour. of the Med. Sciences*) dwells upon the importance of urinalysis as a diagnostic agent of eclampsia. He concludes his article as follows:

1. Careful urinalysis should be carried out in all cases of pregnancy at frequent intervals, and with increased frequency as term is approached.

2. The most dependable indications of impaired renal function and of probable eclampsia have been shown by general experience to be the presence of decided quantities of serum albumin, the diminution of the eliminated urea, and the presence of a microscopic renal sediment (casts, renal epithelium, blood, etc.) The character of the latter, when accompanied by the well known clinical signs of nephritis, always constitutes a working basis for an estimate of the probability of imminent danger.

3. Even if the urine appears perfectly normal the possibility of eclampsia must be considered, especially in young women. Eclampsia in such cases is of equal severity with that of cases in which the urine was given due warning of impaired renal functions.

4. When eclampsia supervenes upon labor in a subject with previously (apparently) healthy kidneys, the tendency subsequently is toward a return to normal renal functions if the patient survives. This circumstance would seem to indicate still more strongly that the kidneys may actually have been normal up to the time of a temporary embarrassment and suspension of function.

5. Until the nature and ultimate cause of uremia and eclampsia are more thoroughly understood, it would appear that urinalysis, though not an unerring guide, is our most valuable index of the condition of the kidneys, and our most trustworthy source of information as to danger from such forms of toxemia.

6. The prognosis seems to be vastly improved if eclampsia be combated by generous bleeding followed by venous transfusion with normal salt solution. These measures reduce and dilute the poison in the circulation, and relieve the cardiac distress. Free diaphoresis and purging are of course indicated.—*Medical Standard.*

---

### TREATMENT OF STERILITY.

Dr. Peter Horrocks (*Lancet,* January 9, 1904. Ref., *The Birmingham Med. Review,* March, 1904, quoted in the *Post Graduate,*) says treatment, of course, depends upon what is discovered by the examination.

1. *No treatment* where woman healthy and not been married three years. If still sterile after three years she may then return for advice.

2. *Food and Exercise.*—Simple food and work, or its equivalent of physical exercise. No alcohol or drugs, especially opiates or sedatives. If the patient is fat permanganate of potash, one or two grains in tabloids after food, three times a day, is good. Spare diet is supposed to improve the chances of having a male child.

3. Tonics can be given if the patient is in bad general health.

4. *Change of Environment or Climate.*—A town patient may be sent to the country. Generally a warmer climate is more favorable to fertility, so such countries as India, South Africa, Italy, Madeira, France, Cornwall, and Devonshire, are suitable. Sending patients to hydropathics or spas is often useful.

5. *Specifics.*—This applies to syphilitic parties only, so mercury or mercury and iodid.

6. *Local Treatment.*—This depends upon what is found by examination. Morbid conditions of the vulva, vagina, cervix, and uterus must be dealt with on ordinary lines. Where there is reflex spasm of the vagina with expulsion of semen post coitum, put in a vulcanite pessary and advise incomplete penetration. Stem pessaries and electricity are of little service. When the fallopian tubes or the ovary is the seat of old inflammatory changes it is not justifiable to perform laparatomy for it alone owing to the risk to life. Should the abdomen be opened for any other cause, then the freeing of adhesions and the opening up of a closed fallopian tube might prove of great service.

7. *Regulation of Coitus.*—Remove any source of pain; advise against excess, and recommend a day or two before the aura as the best time. It might be necessary to enforce abstinence for a week to a month or more.

8. *Natural Habits.*—Be sure a vaginal douche is not being used for any supposed vaginitis. Should all measures fail, attention must be turned to the husband as a possible cause of sterility.

